# Aerobic Exercise as a Tool to Improve Hippocampal Plasticity and Function in Humans: Practical Implications for Mental Health Treatment

**DOI:** 10.3389/fnhum.2016.00373

**Published:** 2016-07-29

**Authors:** Aaron Kandola, Joshua Hendrikse, Paul J. Lucassen, Murat Yücel

**Affiliations:** ^1^Brain and Mental Health Lab, School of Psychological Sciences and Monash Institute of Cognitive and Clinical Neurosciences, Monash University, MelbourneVIC, Australia; ^2^Amsterdam Brain and Cognition, University of AmsterdamAmsterdam, Netherlands; ^3^Centre for Neuroscience, Swammerdam Institute of Life Sciences, University of AmsterdamAmsterdam, Netherlands

**Keywords:** aerobic fitness, hippocampus, plasticity, schizophrenia, depression, dementia, memory, neurogenesis

## Abstract

Aerobic exercise (AE) has been widely praised for its potential benefits to cognition and overall brain and mental health. In particular, AE has a potent impact on promoting the function of the hippocampus and stimulating neuroplasticity. As the evidence-base rapidly builds, and given most of the supporting work can be readily translated from animal models to humans, the potential for AE to be applied as a therapeutic or adjunctive intervention for a range of human conditions appears ever more promising. Notably, many psychiatric and neurological disorders have been associated with hippocampal dysfunction, which may underlie the expression of certain symptoms common to these disorders, including (aspects of) cognitive dysfunction. Augmenting existing treatment approaches using AE based interventions may promote hippocampal function and alleviate cognitive deficits in various psychiatric disorders that currently remain untreated. Incorporating non-pharmacological interventions into clinical treatment may also have a number of other benefits to patient well being, such as limiting the risk of adverse side effects. This review incorporates both animal and human literature to comprehensively detail how AE is associated with cognitive enhancements and stimulates a cascade of neuroplastic mechanisms that support improvements in hippocampal functioning. Using the examples of schizophrenia and major depressive disorder, the utility and implementation of an AE intervention to the clinical domain will be proposed, aimed to reduce cognitive deficits in these, and related disorders.

## Introduction

The brain is continuously balancing two conflicting demands: it must retain enough structural integrity to maintain proper neurotransmission, and function efficiently, whilst remaining malleable enough to restructure itself and adapt to changing environmental demands. The dynamic nature of the brain is underpinned by the concept of neuroplasticity, which refers to the brains capacity to change and reorganize itself in response to internal and/or external influences. The impact and eventual consequences of brain plasticity can be twofold; i.e., these influences can be adaptive, such as during skill learning, when they help the individual to survive, or can be maladaptive, when plasticity is insufficient to meet a specific demand, which may then contribute toward disease conditions.

To some extent, brain disorders can be considered maladies of neuroplasticity (Krystal et al., 2009). As such, stimulating neuroplasticity is becoming a popular approach aimed to counteract pathological harm (Kays et al., 2012). Interestingly, a stimulus as peripheral as aerobic exercise (AE) has been demonstrated to have a strong influence on inducing neuroplasticity (Voss et al., 2013a) and promoting cognitive performance (Smith et al., 2010). Given its general benefits to ones physical health, low risk profile and relative ease of implementation, AE represents a promising therapeutic target for a range of brain pathologies.

One brain region with a high degree of endogenous neuroplasticity is the hippocampus (Bavelier and Neville, 2002). The hippocampus is heavily involved in learning and memory processes and is particularly vulnerable to damage in various pathological conditions like major depressive disorder (MDD), Alzheimer’s disease and schizophrenia (Adriano et al., 2012; Bartsch and Wulff, 2015; Schmaal et al., 2016). Conversely, the positive influence of AE on neuroplasticity is more pronounced within the hippocampus than in any other brain region (Gomez-Pinilla and Hillman, 2013). AE has been suggested as a promising approach in remediating hippocampal harm and cognitive deficits caused by neurodegenerative disorders like AD (Intlekofer and Cotman, 2013), but AE may also be a promising approach for various psychiatric disorders like schizophrenia or MDD (Oertel-Knöchel et al., 2014).

This paper will review the cognitive benefits associated with AE and focus on aspects of cognition that are particularly dependent hippocampal functioning such as episodic memory formation. We further discuss the capacity of AE to stimulate macro- and micro-scale neuroplastic mechanisms relating to hippocampal functioning. Finally, we address the suitability of AE to be used as a novel therapeutic intervention for psychiatric disorders. Both schizophrenia and MDD are associated with hippocampal deterioration and cognitive dysfunctions, so we take these two conditions as examples to discuss the potential utility of AE-based interventions.

## Exercise and Cognition

The interest in how AE influences cognitive performance has exploded in the past decade. AE generally refers to exercise that improve the efficiency of aerobic energy producing systems by increasing maximal oxygen uptake and cardiorespiratory endurance (Voss et al., 2013a). Large-scale epidemiological studies have consistently correlated high levels of aerobic fitness with greater academic achievement and IQ scores (Sibley and Etnier, 2003; Tomporowski et al., 2008, 2014; Howie and Pate, 2012) as well as with a greater preservation of cognitive function in old age (Yaffe et al., 2001; Barnes et al., 2003; Middleton et al., 2008; Wendell et al., 2014) and fewer incidences of dementia (Hamer and Chida, 2009). The capacity to promote cognitive performance in this way implies that AE may have an important clinical relevance in counteracting the cognitive decline associated with aging or dementia (Kramer et al., 2006) and has catalyzed its systematic investigation. Many randomized controlled trials (RCTs) have now been conducted using AE interventions of a moderate intensity (such as 30 min of Nordic walking) that generally span for 3–12 months and are mostly conducted in older adults. Meta-analyses have found AE interventions to improve cognitive performance across a variety of domains, including attention, executive functioning, processing speed, motor functioning, and memory in healthy young and middle aged adults (Etnier et al., 1997; Smith et al., 2010; Chang et al., 2012; Roig et al., 2013; Verburgh et al., 2013) but mostly in older age groups (Etnier et al., 1997; Colcombe and Kramer, 2003; Angevaren et al., 2008; van Uffelen et al., 2008; Smith et al., 2010; Snowden et al., 2011; Chang et al., 2012), as well as in older individuals with mild cognitive impairments or dementia (Heyn et al., 2004; van Uffelen et al., 2008; Gates et al., 2013).

The available evidence strongly suggests that AE has a positive influence on cognition in individuals of all age groups, particularly in older adults. However, the exact nature of how AE impacts upon cognition is not yet clear. Some RCTs have stipulated that AE influences divergent cognitive domains, whereas others have suggested AE had no significant impact on cognition at all (Etnier et al., 2006; van Uffelen et al., 2008; Snowden et al., 2011; Gates et al., 2013; Kelly et al., 2014; Young et al., 2015). Such inconsistencies may partially be explained by the methodological variation between RCTs, making it difficult to systematically compare their findings in meta-analyses (Angevaren et al., 2008; Young et al., 2015).

On a neural level, findings have been consistent in demonstrating that AE has a strong, positive influence on the structure of the hippocampus, which is not seen to the same extent in any other brain regions (Gomez-Pinilla and Hillman, 2013). As will be discussed below, it has been extensively demonstrated in animal models that AE stimulates a cascade of neuroplastic mechanisms within the hippocampus that are often paralleled by functional improvements (Voss et al., 2013a; Opendak and Gould, 2015). A large cohort of animal studies have assessed the functional impact of AE using tasks designed to specifically assess hippocampus dependent processing such as spatial (Vaynman et al., 2004) or contextual memory (Radak et al., 2006). Using hippocampus dependent paradigms, AE has been reliably demonstrated to improve task performance in animal models (van Praag, 2009; Voss et al., 2013a), although this does also depend on others aspects of AE such as the duration and intensity of AE (Naylor et al., 2005; Ploughman et al., 2007; O’Callaghan et al., 2009).

Until recently, human studies had primarily focussed on examining the impact of AE on performance in frontal-executive or attentional tasks rather than on specific hippocampus dependent forms of cognition (Ruscheweyh et al., 2011). It is possible that this may have contributed toward the inconsistent findings when investigating the effect of AE on human cognition. Based on the profound impact of AE on the hippocampus, this bottom-up focus on hippocampus dependent processing may also be a useful approach in specifying the impact of AE on human cognition and will be adopted here.

### A Hippocampus-Centric Approach

The hippocampus plays an important role in both learning and memory (Jarrard, 1993) and affective processing (Phillips et al., 2003). The dichotomous functioning of the hippocampus is thought to be reflected in its structure, with affective processing being largely attributed to the ventral hippocampus and learning and memory processes mostly occurring through the dorsal hippocampus (Moser and Moser, 1998). Given that cognition is a central theme of this paper, the following sections will predominantly focus on the role of the hippocampus in learning and memory processing.

Several meta-analyses have denoted the tendency for RCTs to report improvements in memory-based task performance following an AE intervention (Colcombe and Kramer, 2003; van Uffelen et al., 2008; Smith et al., 2010; Chang et al., 2012; Roig et al., 2013). However, some domains of memory are more reliant on hippocampal functioning than others (see **Box 1**) and therefore, it is possible that the most consistent cognitive improvements may be found in these specific domains of memory.

**Hippocampus dependent memory.**

The human hippocampus plays a vital role in the formation of declarative memories, most prominently of which, in the formation of episodic and spatiotemporal memories (Burgess et al., 2002). Episodic memory refers to the recollection of autobiographical events and is related to spatial memory, which refers to one’s environment and spatial orientation. Spatial and episodic memory processes are inherently related given their specific reliance on the hippocampus (Bird and Burgess, 2008) and the fact that episodic memories are encoded in a spatio-temporal context (Tulving, 1993), making spatial information important in episodic memory formation. Also, the hippocampus, and particularly the DG, is crucial in selecting and separating similar events in space and time, and hence pattern separation is a main function attributed to the hippocampus (Yassa and Stark, 2011; Oomen et al., 2013, 2014). It is important to note that given the requirement of a conscious experience to form an episodic memory, at present episodic memory cannot be directly studied in animals given the lack of behavioral markers for their conscious experience (Clayton et al., 2001). Contextual memory is a process strongly related to episodic memory that is also highly dependent on the hippocampus and refers to the capacity for an animal to make associations with salient landmark objects and their environmental context (Eichenbaum et al., 2005). As there is currently no objective proxy for studying episodic-like memory processing in animals (Templer and Hampton, 2013), hippocampal functioning shall be considered here as a function of contextual and spatial memory task performance when referring to animal literature and episodic and spatial memory task performance in human literature.

Some human studies have focussed on assessing the influence of AE on hippocampus dependent cognition and shown in older adults that AE was associated with improved performances in both episodic (Richards et al., 2003; Stewart et al., 2003; Sabia et al., 2009; Flöel et al., 2010; Ruscheweyh et al., 2011) and spatial (Erickson and Kramer, 2009; Erickson et al., 2010, 2011) memory tasks as well as in pattern separation tasks in young adults (Déry et al., 2013). Moreover, some studies have demonstrated in preadolescent children and young adults that AE is selectively associated with improved performances on contextual (Chaddock et al., 2010, 2011; Monti et al., 2012) and spatial (Stroth et al., 2009; Herting and Nagel, 2012) memory tasks and not with less hippocampus dependent tasks such as attention, verbal memory, or item recognition tasks.

Despite a limited selection of studies, these findings indicate that AE may have a positive influence on hippocampus dependent forms of cognition in healthy human participants, similar to what has been consistently shown in animal models. Pertaining to its highly neuroplastic nature (Bavelier and Neville, 2002), the hippocampus is particularly vulnerable to structural and functional deterioration in a wide range of neurological and psychiatric disorders (Bartsch and Wulff, 2015). The aforementioned studies demonstrate that AE could have a positive influence on hippocampal functioning, but a significantly greater cohort of systematic investigations using human participants will be necessary to outline this relationship on a broader cognitive level. A growing body of evidence is also accumulating to suggest that AE may have a prominent impact on hippocampal structure in humans as well as in animal models. The following sections will first discuss the relationship between AE and gross structural changes related to the hippocampus in humans. We will then discuss both animal and human work, which indicates that these structural changes may be driven by a cascade of micro-scale neuroplastic mechanisms within the hippocampus that are stimulated by AE.

## Macro-Scale Changes

Directly studying structural changes in the human brain *in vivo* is currently limited to the use of neuroimaging techniques, like magnetic resonance imaging (MRI), to detect macro-scale changes such as in grey-matter volume or white-matter integrity.

### Gray-Matter

A number of cross-sectional studies have used both voxel-based morphometric (VBM) and region of interest (ROI) techniques on structural MRI data to estimate volume changes associated with AE. Higher levels of aerobic fitness have been consistently associated with larger hippocampal or temporal lobe volumes in healthy adolescent (Chaddock et al., 2010) and older adults (Colcombe et al., 2003; Bugg and Head, 2011; Head et al., 2012; Niemann et al., 2014). Several studies have also shown that hippocampal growth induced by AE correlates with a greater performance on spatial memory tasks such as a virtual Morris Water Maze task (Erickson and Kramer, 2009; Szabo et al., 2011; Herting and Nagel, 2012) and on contextual memory tasks (Chaddock et al., 2010) with correlations ranging from *r* = 0.12 to *r* = 0.36. Cross-sectional data has also indicated that AE may also be beneficial to non-healthy individuals as higher levels of aerobic fitness have also been correlated with larger hippocampal volumes in patients with obesity (Bugg et al., 2012), anorexia (Beadle et al., 2015), mild cognitive impairments (Gates et al., 2013; Makizako et al., 2014), MDD (Travis et al., 2015), Alzheimer’s disease (Honea et al., 2009), and multiple sclerosis (Prakash et al., 2010; Motl et al., 2015).

Several RCTs have extended upon these findings in healthy and non-healthy samples, which have mostly implemented AE interventions of a moderate intensity for 3–12 months. RCTs in healthy samples have demonstrated that AE interventions were associated with increases in hippocampal volume in young and middle aged (Thomas et al., 2016) and older adults (Colcombe et al., 2006; Erickson et al., 2011; Niemann et al., 2014; Kleemeyer et al., 2015; Maass et al., 2015; Sexton et al., 2015a). In some cases the AE induced growth in hippocampal volume was correlated with improved performances on a computerized spatial memory task (*r* = 0.28; Erickson et al., 2011) or on a complex figure test of spatial object recognition (*r* = 0.37; Maass et al., 2015). AE interventions have also been shown to increase hippocampal volume in patients with mild cognitive impairments (ten Brinke et al., 2015), schizophrenia (Pajonk et al., 2010), and multiple sclerosis (Leavitt et al., 2014). While some RCTs have found that AE did not have an impact on hippocampal volume (Ruscheweyh et al., 2011; Scheewe et al., 2013; Krogh et al., 2014; Rosenbaum et al., 2015; Malchow et al., 2016), it is possible that such discrepancies are caused by a lack of consistency in AE protocols used (Prakash et al., 2015) or in the methods of calculating hippocampal volume (Niemann et al., 2014).

### White-Matter

The majority of the current literature has focussed on assessing gray-matter changes, but some studies have investigated the impact of AE on white-matter integrity. A recent systematic review concluded that AE was associated with global and localized improvements in white-matter volume and microstructural integrity in older adults (Sexton et al., 2015b). It would be reasonable to expect AE to have a specific impact on hippocampal connectivity, but such findings were not supported in this review (Sexton et al., 2015b). However, some studies have found AE to be associated with greater white-matter volume in the temporal lobes that surround the hippocampus in older adults (Colcombe et al., 2003, 2006; Tseng et al., 2013; Burzynska et al., 2014). In addition, one RCT that assessed a 12-month AE intervention did demonstrate that greater changes in aerobic fitness were associated with greater improvements in temporal lobe white-matter integrity in healthy, older adults (Voss et al., 2013b). White-matter alterations may also occur in non-healthy individuals as one recent RCT demonstrated a 6-month AE intervention to improve global white-matter integrity in patients with schizophrenia (Svatkova et al., 2015).

### Structural Benefits of AE

There is a growing cohort of evidence to suggest that AE is associated with increases in hippocampal gray-matter volume in both healthy and non-healthy individuals (Hötting and Röder, 2013; Hamilton and Rhodes, 2015), and with increases in hippocampal microstructural integrity (Kleemeyer et al., 2015). In some cases, these AE-induced gray-matter changes have been directly correlated with improvements in hippocampal functioning (Prakash et al., 2015). AE appears to have a beneficial impact on global white-matter integrity (Sexton et al., 2015b) and potentially on hippocampal connectivity.

These studies support the idea that AE may be beneficial to hippocampal integrity but it is important to note that these human imaging techniques may only directly assess macro-scale changes and not the functional changes or biological mechanisms that may underlie these changes. Without information relating to the specific substrates underlying changes in tissue composition, it is difficult to determine the exact functional significance of a change in volume (Czéh and Lucassen, 2007; Thomas et al., 2012; Biedermann et al., 2016). For example, volume growth could be driven by a regional increase in dendritic length or density, but it could also be driven by changes that are likely to be less functionally relevant, such as expanding interstitial space between cells or changes in relative water distributions. Alternatively, increases in the proliferation of stem cells, glia, or the birth of new neurons that are added to existing hippocampal circuitry could also influence hippocampal volume over time. Furthermore, the additional energy demands from new neurons or synaptic changes may also require greater metabolic support. This could induce gliogenesis or changes in vasculature, i.e., angiogenesis, that may further contribute to fluctuations in volume (Anderson, 2011). In many respects, such structural adaptations resemble some of the opposite changes seen after exposure to (chronic) stress, that are generally associated with hippocampal volume reductions and represents a risk factor for depression (Czéh and Lucassen, 2007). Whether the same substrates underlie both the atrophy and the growth of a brain region is not clear and in principle, different mechanisms may be responsible for such opposing effects.

Micro-scale changes on a molecular or cellular level can be directly studied using histological approaches in animal models *in vivo*. Therefore, the following section will focus predominantly on animal literature to outline the micro-scale impact of AE on hippocampal neuroplasticity that may drive these macro-scale structural and functional improvements seen in human studies.

## Micro-Scale Changes

Aerobic exercise has been linked to changes in a range of independent and interdependent mechanisms of neuroplasticity within the hippocampus (for comprehensive reviews, see: van Praag, 2009; Gomez-Pinilla and Hillman, 2013; Voss et al., 2013a; Bolijn and Lucassen, 2015; Opendak and Gould, 2015). Key mechanisms at both the cellular and molecular level will be discussed below in terms of their contribution to AE-induced enhancements in hippocampal functioning.

### Neurogenesis

Adult neurogenesis is a form of structural hippocampal plasticity that refers to the process of stem cells forming new neurons within a few, distinct sub-regions of the adult brain. These stem cells undergo subsequent stages of proliferation, migration and neuronal differentiation, eventually producing adult-generated, fully functional, neurons that are well integrated into existing neural circuits (Kempermann et al., 2015). The subgranular zone of the hippocampal dentate gyrus (DG) is one of the just two primary locations where neurogenesis is known to occur in the adult rodent and human brain (Spalding et al., 2013; Kempermann et al., 2015). AE increases the rate of hippocampal neurogenesis, i.e., both the rate of cell proliferation as well as the survival of newborn granule cells (Cotman et al., 2007; Kempermann et al., 2010; Lucassen et al., 2010; Vivar et al., 2013) and despite being difficult to study in humans (Manganas et al., 2007; Ho et al., 2013; Jessberger and Gage, 2014), AE may also stimulate cell proliferation and DG volume growth in the human brain (Pereira et al., 2007; Erickson et al., 2011; Demirakca et al., 2014).

The proliferation of adult-born granule cells is thought to play an important role in hippocampal functioning (Deng et al., 2010; Oomen et al., 2014). It has been extensively demonstrated that the process of neurogenesis modifies the excitation of hippocampal neurons (Ikrar et al., 2013). Animal models have demonstrated that the inhibition or ablation of neurogenesis impairs performance on spatial and contextual memory tasks while improved performances on these tasks are seen when neurogenesis is stimulated (Deng et al., 2010; Snyder and Cameron, 2012; Aimone et al., 2014; Vadodaria and Jessberger, 2014; Kent et al., 2015). Furthermore, manipulating the rate of proliferation in rodents has been shown to selectively effect hippocampus dependent task performance, such as in spatial and contextual memory or pattern separation tasks, but not in tasks that are less hippocampus dependent, such as delay conditioning (Gould et al., 1999; Shors et al., 2002; Snyder et al., 2005, 2011; Deng et al., 2009; Sahay et al., 2011; Lucassen et al., 2013).

While there is still debate as to the exact role that adult-born granule cells play in hippocampal functioning, the process of neurogenesis forms a substrate for experience-dependent change (Opendak and Gould, 2015; Lucassen and Oomen, 2016), which has been implicated in fear and anxiety and depression like-behavior (Sahay and Hen, 2007; Besnard and Sahay, 2015; Lucassen et al., 2015; Hu et al., 2016). This primarily occurs through the role that neurogenesis plays in facilitating memory formation by dictating a computational mechanism known as *pattern separation* (Aimone et al., 2011; Sahay et al., 2011). Pattern separation is an essential mechanism for the DG to efficiently process and store sensory inputs to enable the formation of episodic, contextual or spatial memories (Lazarov and Hollands, 2016), and its importance in AE induced cognitive enhancement is epitomized by correlative studies that manipulate neurogenesis (Kent et al., 2015; Lucassen and Oomen, 2016). For example, inhibiting the rate of cell proliferation in mice was sufficient to impair pattern separation (Deng et al., 2010) and block AE-induced improvements in spatial (Clark et al., 2009) and contextual (Wojtowicz et al., 2008) memory performance, but only in conditions in which the task required fine spatial discriminations (i.e., where pattern separation was necessary; Creer et al., 2010). Therefore, AE may augment hippocampal memory formation by minimizing the interference between highly similar inputs through increasing the rate of cell proliferation (Déry et al., 2013).

While pattern separation is a popular example of DG function, adult-born granule cells have also been associated with a number of other theoretical DG frameworks important to memory formation that are not discussed here, such as memory resolution or encoding temporal context (see Aimone et al., 2014 for a comprehensive review). While neurogenesis is a crucial process to the spatiotemporal aspect of hippocampal functioning and is likely to be a key mediator of the hippocampal enhancement stimulated by AE (Cotman et al., 2007), more research is needed to elucidate the exact role that adult-born granule cells play in DG and general hippocampal functioning.

Stimulating neurogenesis and promoting DG function is one mechanism through which AE may promote hippocampal function and some recent findings suggest this mechanisms may be driving the macro-scale increases in hippocampal volume found in human imaging studies (Erickson et al., 2011; Fuss et al., 2014). However, AE also induces a variety of other neuroplastic mechanisms that work both independently and in tandem with neurogenesis to improve hippocampal functioning.

### Synaptic Plasticity

Learning and memory is reliant upon the efficient communication between neural cells through their synapses and AE is thought to enhance this efficiency through promoting synaptic plasticity in a number of ways (Vivar et al., 2013), such as through facilitating long-term potentiation (LTP). LTP is one model of synaptic plasticity that refers to the strengthening of synaptic connections between neurons (Bliss and Collingridge, 1993). Forming an episodic memory involves the association of an event or feature with a particular location in space and occurs through this LTP mechanism in the hippocampus (Bannerman et al., 2014). AE has been demonstrated to stimulate LTP in young rodents and reverse the age-related decline of LTP within the DG of aged rodents compared to sedentary controls (van Praag, 2009; Voss et al., 2013a). Interestingly, these changes appear to be specific to the DG region and may be directly related to the stimulation of neurogenesis by AE (van Praag, 2008). Indeed, immature granule cells are particularly suited to participate in the learning process as they have a lower threshold for LTP induction (Schmidt-Hieber et al., 2004) and demonstrate enhanced LTP compared to surrounding mature granule cells (Lee S.W. et al., 2012). Being hypersensitive to influence on their synaptic plasticity has led to the idea that these adult-born granule cells mediate the enhancement of LTP in the DG from AE (Voss et al., 2013a).

Aerobic exercise has also been associated with certain morphological changes to the structure of neural cells, which may be important in facilitating hippocampal learning and memory (Lang et al., 2004). The dendrites of granule cells within the DG have been shown to increase in length, complexity, and spine density in response to AE (Eadie et al., 2005; Redila and Christie, 2006) as well as spine density in surrounding pyramidal cells of the entorhinal cortex and CA1 regions (Stranahan et al., 2007). AE also interacts with the glutamatergic system through increasing the expression of *N*-methyl-D-aspartic acid (NMDA) receptors in the hippocampus (specifically, both NR2A and NR2B subtypes), which contributes toward the synaptic plasticity of the region (Molteni et al., 2002; Farmer et al., 2004). These morphological changes are associated with higher rates of LTP induction and facilitate changes in dendritic strength (van Praag, 2008).

While certain morphological changes enhance synaptic plasticity within the CA1 and entorhinal cortex, AE seems to have a particularly potent impact on the granule cells of the DG. Through generating a greater influx of adult-born granule cells that have an enhanced propensity for LTP and fine-grained morphological changes, AE stimulates an environment within the hippocampus that promotes LTP and facilitates improvements in hippocampus-dependent cognition (Boecker et al., 2012).

### Vasculature

Cerebral blood flow is important in providing oxygen and essential nutrients that facilitate brain functioning. Improving cerebral blood flow may be an important mediator of AE induced changes in hippocampal functioning (Christie et al., 2008). It has been demonstrated in animal models that AE stimulates the sprouting of new capillaries (angiogenesis) and improves vasculature within the hippocampus (Trejo et al., 2001; van Praag et al., 2005). This coincides with studies that have demonstrated a greater cerebral blood flow in the human hippocampus (Pereira et al., 2007; Burdette et al., 2010; Maass et al., 2015), some of which have correlated this increase with improved performance on episodic memory tasks (Pereira et al., 2007; Maass et al., 2015). The positive influence of improving vascularization may extend beyond a greater supply of oxygen and glucose through prompting the release of neurotrophic factors (the influence of which will be discussed below; Maass et al., 2015) or through facilitating other neuroplastic mechanisms like synaptic plasticity (Christie et al., 2008) or neurogenesis (Palmer et al., 2000; Pereira et al., 2007; Boecker et al., 2012; Bolijn and Lucassen, 2015; Biedermann et al., 2016). In fact, one recent human RCT found that the improvements in hippocampus dependent task performance and growth in hippocampal volume, which occurred following a 3-month AE intervention were predominantly attributable to a greater cerebral blood flow in the hippocampus (Maass et al., 2015). The authors suggested that these changes were either a direct consequence of vascularization or an indirect consequence of changes in synaptic plasticity or neurogenesis that were stimulated by the improvements in cerebral blood flow.

Quantifying the impact that changes in vasculature induced by AE have on hippocampal functioning is increasingly attracting attention, but the relative contribution of changes in vasculature is still in contention. For example, some animal models have suggested that angiogenesis may underlie improvements in spatial memory tasks independent of other neuroplastic mechanisms (van Praag et al., 2007; Kerr et al., 2010), whereas others have found improvements in spatial learning to be driven by neurogenesis with no influence at all from angiogenesis (van Praag et al., 2005). Changes in vasculature may indeed stimulate other neuroplastic mechanisms like synaptogenesis or neurogenesis and promote hippocampal tissue growth (Kleemeyer et al., 2015), but a greater investigation is required to outline the direct relationship between vascularization and enhancements in hippocampal functioning stimulated by AE (Davenport et al., 2012). Interestingly, AE is specifically associated with an increase the density of small, rather than large-diameter blood vessels in both humans (Bullitt et al., 2009) and animals (Bloor, 2005; Van der Borght et al., 2009). Increasing microvasculature density may be protective against white-matter hyperintensities, which itself may reduce gray-matter atrophy and cognitive dysfunction (Voss et al., 2013a). Therefore, in additional to promoting hippocampal function, the influence of AE on vasculature may also preserve structural integrity in the hippocampus and other regions of the brain.

On a cellular level, both animal and human literature has suggested that neurogenesis, synaptic plasticity and vascularization within the hippocampus represent the three primary neuroplastic mechanisms that are stimulated by AE to promote hippocampal functioning. These cellular changes are in turn influenced by a number of changes that occur on a molecular level in response to AE and the relevance of these molecular factors to hippocampal functioning will be considered below.

### Neurotrophic Factors

Neurotrophins are important to the development and maintenance of functioning neural cells in the brain (Barbacid, 1995) and are likely to play a crucial role in mediating the impact of AE on hippocampal neuroplasticity and functioning (Cotman et al., 2007; Voss et al., 2013a; Bolijn and Lucassen, 2015). Brain-derived neurotrophic factor (BDNF) is a centrally produced neurotrophin of particular interest due to its high concentration within the hippocampus and its integral role in supporting neuronal survival and growth, and synaptic plasticity (Cotman et al., 2007; Cowansage et al., 2010). Animal models have shown AE to be associated with a region-specific up-regulation of BDNF in the hippocampus (Neeper et al., 1995; Marlatt et al., 2012; Uysal et al., 2015). Similarly, in human models AE has been associated with an increase in BDNF serum (Coelho et al., 2013) with some indication of this up-regulation occurring specifically within the hippocampus (Erickson et al., 2011; Voss et al., 2013a).

Brain-derived neurotrophic factor may be the most important factor that is upregulated by AE given its extensive involvement in both synaptic plasticity and neurogenesis (Cotman et al., 2007). The protein is thought to interact with energy metabolism to facilitate both pre- and post-synaptic mechanisms and the induction of LTP (Gomez-Pinilla and Hillman, 2013; Edelmann et al., 2014), as well as promoting the proliferation and survival of adult-born granule cells (Korol et al., 2013; Park and Poo, 2013). The importance of BDNF to hippocampal neuroplasticity is epitomized by animal studies, which have found that the manipulation of BDNF expression directly impacts upon performance in spatial and contextual memory tasks (Linnarsson et al., 1997; Alonso et al., 2002; Heldt et al., 2007; Peters et al., 2010), and also that the downregulation of BDNF inhibits AE induced improvements in spatial memory performance (Vaynman et al., 2004).

Recently, a number of human studies have also demonstrated that the increased BDNF serum levels associated with AE are directly correlated with improved performances in various domains of memory including on spatial memory tasks (Piepmeier and Etnier, 2015). In line with the molecules’ interaction with neurogenesis and particularly high concentration within the DG subfield of the hippocampus (Farmer et al., 2004), BDNF may play an important role in pattern separation. Recent studies have shown that BDNF is required for the pattern separation to occur in memory encoding and consolidation, and specifically that BDNF acts on adult-born granule cells within the DG to facilitate pattern separation (Bekinschtein et al., 2013, 2014).

Vascular endothelial derived growth factor (VEGF) and insulin growth factor 1 (IGF-1) are growth factors that are produced peripherally and are implicated in both angiogenesis and neurogenesis (Gomez-Pinilla and Hillman, 2013). Human studies have shown that AE is associated with peripheral increases in VEGF and IGF-1 serum concentrations (Schobersberger et al., 2000; Llorens-Martín et al., 2010), and both molecules are known to cross the blood brain barrier and interact with hippocampal cells (Ding et al., 2006; Tang et al., 2010). VEGF is a hypoxia-inducible protein that stimulates angiogenesis (Krum et al., 2002). By facilitating vascularization in this way, particularly within the DG (Clark et al., 2009), the molecule also supports the AE induced stimulation of neurogenesis (Gomez-Pinilla and Hillman, 2013). Similarly, IGF-1 is known to support alterations in vascularization that are induced by AE (Lopez-Lopez et al., 2004) and promotes the proliferation and survival of newborn neural cells (Gomez-Pinilla and Hillman, 2013). Indeed, the inhibition of VEGF or IGF-1 has been demonstrated to impair the promotional impact of AE on the rate of neurogenesis (Trejo et al., 2001; Fabel et al., 2003; Ding et al., 2006) and spatial memory performance (Ding et al., 2006). Conversely, up-regulating either VEGF or IGF-1 can stimulate neurogenesis independently of AE (Aberg et al., 2000; Carro et al., 2000; Trejo et al., 2001; Cao et al., 2004).

While a number of other biochemical changes occur in response to AE (see Bolijn and Lucassen, 2015), BDNF, VEGF, and IGF-1 are considered to be key proteins upregulated by AE that induce hippocampal neuroplasticity. The important contribution that these neurotrophins make to the neuroplasticity induced by AE is underscored by animal models, which demonstrate their direct and significant impact on hippocampal functioning (Cotman et al., 2007; Llorens-Martín et al., 2010; Voss et al., 2013a; Piepmeier and Etnier, 2015). Subsequently, human studies are starting to take a step further than only assessing the impact of AE in terms of macro-scale structural changes by attempting to indirectly study underlying biological processes.

### Inferring Micro-Scale Changes Using Human Imaging

An important approach has been to supplement volumetric changes with recordings of peripheral biomarkers as a proxy for measuring changes in neurotrophin regulation within the brain. Results using this approach have thus far have been mixed, with some AE studies findings a positive correlation between hippocampal volume or connectivity changes and serum concentrations of BDNF, VEGF, and IGF-1 (Erickson et al., 2011; Coelho et al., 2013; Voss et al., 2013b), while others have failed to replicate such findings (Maass et al., 2016).

Another promising approach has been to combine multimodal imaging techniques to estimate the micro-scale processes that underlie volumetric changes associated with AE. For example, one study demonstrated that AE stimulated hippocampal volume growth and then utilized a range of multi-modal MRI techniques to indirectly suggest that this growth may be supported by changes in myelination, rather than in vasculature (Thomas et al., 2016). Some studies have used diffusion tensor imaging (DTI) to demonstrate that the growth in hippocampal volume induced by AE is correlated with improvements in the microstructural integrity of hippocampal gray-matter, based on the assumption that a low mean diffusivity is indicative of an increased gray-matter tissue density (Kleemeyer et al., 2015). Another multimodal approach that is growing in popularity is the use of MR spectroscopy (MRS) to measure microscale changes in local metabolite composition. *N*-acetylaspartate (NAA) is a metabolite indicative of neuronal integrity and a number of AE studies have demonstrated that the growth in hippocampal volume (Pajonk et al., 2010; Erickson et al., 2012; Gonzales et al., 2013; Wagner et al., 2015) and in some cases, improvements in working memory performance (Erickson et al., 2011) are both correlated with higher concentrations of NAA. One of these studies found that an AE intervention led to a 2% decrease in hippocampal volume but the MRS data indicated there was no change in NAA (Wagner et al., 2015). Therefore, given the approximation that 50% of gray-matter is composed of neuropil (Thomas et al., 2012), the authors suggested that the volumetric decline is unlikely to have resulted from a loss of neurons (which would have been indicated by a lower NAA value) and may be due to other factors such as changes in glial cells (Wagner et al., 2015). This analysis and others (Dennis et al., 2015) illustrate the utility of using multimodal imaging such as MRI with MRS to assess the functional relevance of volumetric alterations that is not possible using one modality alone. Future research would benefit from adopting methods similar to those outlined above to aid in the translation between animal and human models and help provide a more comprehensive account detailing the impact of AE on the human brain.

Animal studies have demonstrated that AE stimulates a cascade of interdependent cellular and molecular mechanisms of neuroplasticity that mediate the associated enhancements in hippocampal functioning. In humans, AE is associated with increases in hippocampal volume and some indirect indicators of neuroplasticity (e.g., increased cerebral blood flow or BDNF serum concentration), which correlate with improved performances on hippocampus dependent tasks, indicative of an enhanced hippocampal functioning. These findings suggest that AE can stimulate hippocampal neuroplasticity and promote the regions functioning in humans in a similar way to that which has been demonstrated using animal models. Considerably more research will be necessary to substantiate this relationship and methods such as multimodal imaging and assessing peripheral biomarkers represent promising ways that future RCTs can help to bridge the gap between animal and human models. Based on the available evidence outlined above, AE does appear to have a highly beneficial impact on hippocampal integrity and the following sections will discuss whether AE thus represents a viable clinical intervention.

## Clinical Application

The capacity for AE to induce neuroplastic changes that improve both hippocampal integrity and promote hippocampus dependent cognition may be of particularly clinical importance for two reasons. Firstly, a number of psychiatric and neurological disorders seem to have a particularly potent influence on hippocampal structure (Bartsch and Wulff, 2015) and its deterioration may underlie certain aspects of their symptomatology. Secondly, psychiatric symptoms can be dichotomously described as being either affective or cognitive in nature, yet a disproportionate amount of the current literature has exclusively focussed on ameliorating affective symptoms (Millan et al., 2012). Currently, no effective treatments have been developed to alleviate cognitive deficits associated with psychiatric or neurological disorders (Wallace et al., 2011; Keefe et al., 2013, 2014; Solé et al., 2015). Promoting neuroplastic changes that enhance hippocampal functioning may be useful in remediating certain domains of cognitive dysfunction (e.g., in learning and memory), which occur in disorders that have a particularly detrimental impact on hippocampal integrity. The efficacy of AE is already being investigated as a therapeutic intervention to counteract cognitive decline and deteriorating hippocampal integrity associated with aging or neurological disorders like dementia (Ahlskog et al., 2011). Comparatively little attention has been afforded to the potential application of AE in treating psychiatric disorders that have a similarly deleterious impact on the hippocampus and are associated with severe cognitive deficits. The following sections will outline the need to develop effective interventions that remediate cognitive symptoms, which can have a debilitating impact on psychiatric patients, using the examples of schizophrenia and MDD. The capacity for AE to aid in the alleviation of cognitive symptoms and improve the efficacy of current treatment will then be discussed in the context of both MDD and schizophrenia.

### Cognitive Dysfunction in Psychiatric Disorders

Behaviors observed in animal models of anxiety/MDD have indicated that MDD can cause deficits in several areas of cognition, many of which are strongly related to hippocampal function (Darcet et al., 2016). A number of animal studies have shown models of anxiety/MDD to be associated with poorer performances on tasks of working memory (Mizoguchi et al., 2000; Henningsen et al., 2009) and attention (Baudin et al., 2012; Wilson et al., 2012; Wallace et al., 2014), as well as on tasks reliant on hippocampal functioning such as episodic-like memory (Orsetti et al., 2007; Baudin et al., 2012; Naninck et al., 2015) and spatial memory (Markham et al., 2010; Darcet et al., 2014). Similarly, human patients with MDD have consistently demonstrated clinically relevant deficits in domains of executive functioning, psychomotor speed, attention, and memory (McDermott and Ebmeier, 2009; Lee R.S. et al., 2012; McIntyre et al., 2013; Rock et al., 2013). Even after remission following successful antidepressant treatment, most patients continue to experience cognitive deficits, particularly within domains of executive function, memory, and attention (Hammar and Ardal, 2009; Bora et al., 2013; Rock et al., 2013; Popovic et al., 2015; Solé et al., 2015). Individuals with MDD who continue to experience these cognitive symptoms are more likely to relapse and display worse psychosocial functioning outcomes (Papakostas et al., 2008; Bortolato et al., 2016), which can impact their capacity to socialize or to function well at work (Jaeger et al., 2006; McIntyre et al., 2013). Consequentially, the importance of alleviating cognitive dysfunction to enhance the success of MDD treatment is increasingly being recognized (Solé et al., 2015; Bortolato et al., 2016).

The heterogeneity of schizophrenia makes the disorder extremely difficult to model in animals and most approaches have focussed on replicating certain groups of schizophrenia-like behaviors in animals (Nestler and Hyman, 2010). For example, disrupting NMDA receptors with phencyclidine (PCP) is known to produce a range of symptoms associated with schizophrenia (Nestler and Hyman, 2010) and has subsequently been shown to cause a number of cognitive deficits including in cognitive flexibility (Abdul-Monim et al., 2007), attention (Amitai et al., 2007) and episodic-like memory (Grayson et al., 2007; Nagai et al., 2009). Cognitive dysfunction in human patients with schizophrenia is rapidly being considered amongst the most debilitating aspects of schizophrenia (Nuechterlein et al., 2011) and the severity of cognitive dysfunction is a key determinant of the functional outcome following treatment (Goldberg and Green, 2002). Schizophrenia is associated with deficits in cognitive domains of executive functioning, processing speed, memory and attention (Mesholam-Gately et al., 2009; Keefe and Harvey, 2012). The persistence of these deficits has a significant impact on an individual’s quality of life and ability to attain and maintain employment (Archer and Kostrzewa, 2015) making the amelioration of cognitive deficits a highly prioritized therapeutic goal in schizophrenia treatment (Malchow et al., 2013).

### The Underlying Neuroplasticity of Cognitive Dysfunction

Both MDD and schizophrenia have a detrimental impact on neuroplasticity, particularly within the hippocampus. A reduced hippocampal volume is one of the most consistently reported structural abnormalities in patients of MDD (Schmaal et al., 2016) and of schizophrenia (Ellison-Wright and Bullmore, 2010; Adriano et al., 2012). In patients with schizophrenia, these hippocampal abnormalities have been correlated with deficits in memory (Gur et al., 2000) and aspects of executive functioning involving inhibitory control (Bilder et al., 1995; Szeszko et al., 2002), demonstrating the importance of hippocampal abnormalities to cognitive dysfunction. Both disorders seem to have a particularly deleterious impact on DG volume (Tamminga et al., 2010; Huang et al., 2013; Travis et al., 2015), which is reminiscent of the reduced level of neurogenesis found in animal models of both disorders (Eisch and Petrik, 2012; Lucassen et al., 2015; Allen et al., 2016). Schizophrenia and MDD also inhibit other important neuroplastic mechanisms such as synaptic plasticity (Law and Deakin, 2001; Kolomeets et al., 2007; Kobayashi, 2009; McEwen et al., 2012; Sanderson et al., 2012) and BDNF expression (Krishnan and Nestler, 2008; Green et al., 2011; Favalli et al., 2012) within the hippocampus. The inhibition of such key neuroplastic mechanisms in the hippocampus is likely to be an important factor contributing toward the cognitive deficits associated with MDD (Perera et al., 2008; Kaymak et al., 2010; Nagahara and Tuszynski, 2011; Turner et al., 2012) and schizophrenia (Ranganath et al., 2008; Schobel et al., 2009; Heckers and Konradi, 2010; Zhang et al., 2012).

Targeting neuroplasticity has recently become an important approach in treating psychiatric symptoms (Kays et al., 2012) and may be useful in counteracting this hippocampal harm to remediate cognitive deficits. For example, deficits in episodic memory have been consistently found in patients with MDD (McDermott and Ebmeier, 2009) and schizophrenia (Barch and Ceaser, 2012), and given the role of the hippocampus in episodic memory formation this may be somewhat attributable to its dysfunction. Indeed, aspects of hippocampal dysfunction such as a lower basal rate of neurogenesis are thought to inhibit one’s capacity for pattern separation in patients with MDD (Déry et al., 2013; Shelton and Kirwan, 2013) or schizophrenia (Das et al., 2014) and the negative impact that this has on episodic memory formation may have broad implications for the symptomology of both disorders (Dere et al., 2010; Tamminga et al., 2010).

To illustrate this point, deficits in pattern separation could negatively influence one’s capacity to correctly discriminate between stimuli and lead to a tendency for overgeneralization (Kheirbek et al., 2012; Shelton and Kirwan, 2013). The pre-disposition for individuals with MDD to make negative self-inferences coupled with the tendency to overgeneralize could result in negative overgeneralizations in the formation of episodic memories, which may contribute toward affective symptoms such as anhedonia (Eisch and Petrik, 2012; Shelton and Kirwan, 2013). In the case of schizophrenia, the added complication of a reduced synaptic connectivity between the DG and CA3 subfields (Kolomeets et al., 2007) to a lower basal rate of neurogenesis may contribute toward psychosis to some extent. Patients with schizophrenia show a disproportionately low level of pattern separation relative to pattern completion (Tamminga et al., 2010). Pattern completion is a complimentary process to pattern separation whereby through the activation of associative networks in the CA3 region, partial information can act as a recall cue to return the full representation of a previously stored memory (Nakashiba et al., 2012). It is possible that this overactivation of the pattern completion mechanism could lead to the inappropriate associations and representations causing the encoding or retrieval of false episodic memories with psychotic content (Tamminga et al., 2010; Das et al., 2014).

These two theoretical examples illustrate how hippocampal dysfunction in either disorder may disrupt the correct formation of episodic memories and potentially exasperate other psychiatric symptoms. Within this framework, the stimulation of neuroplastic mechanisms like neurogenesis might help to alleviate deficits in episodic memory, subsequently help to reduce the expression of other psychiatric symptoms. Furthermore, given the regions importance to learning and memory processing, it is possible that improving hippocampal functioning would also contribute toward the alleviation of other cognitive deficits than episodic memory. For example, deficits in working memory represent an aspect of executive functioning impaired in MDD (Lee R.S. et al., 2012; Rock et al., 2013) and schizophrenia (Forbes et al., 2009). As hippocampal functioning may be an important factor supporting working memory (Fell and Axmacher, 2011; Chaieb et al., 2015) promoting hippocampal integrity may indirectly contribute toward alleviating working memory deficits and improving executive functioning.

While it has not been the focus of this paper, it is also worth noting that altered hippocampal functioning can directly influence other, non-cognitive processes that are important in the pathology of certain psychiatric disorders like MDD. Through its dense connectivity with the prefrontal cortex and the amygdala, the hippocampus is also implicated in emotional regulation (O’Donnell and Grace, 1995; Seidenbecher et al., 2003; Lisman and Grace, 2005; Maren and Hobin, 2007) as well as playing an important role in regulating feedback inhibition from the hypothalamic-pituitary-adrenal axis (Jacobson and Sapolsky, 1991). Hippocampal dysfunctioning may therefore contribute toward deficits in the regulation of emotional processing and stress responses that are often seen in patients with MDD (Sapolsky, 2000; Davidson et al., 2002).

The role of these deficits in neuroplasticity within the hippocampus may be a crucial factor underlying hippocampal dysfunction in psychiatric disorders like MDD and schizophrenia. Directly targeting these deficits to enhance hippocampal functioning may help to improve deficient cognitive processes whose underlying etiology are particularly localized to the hippocampus. Furthermore, it is possible that promoting hippocampal functioning may have a beneficial impact on alleviating other cognitive or affective symptoms, which are more broadly related to dysfunctional networks that involve the hippocampus.

### Current Treatment Approaches

Psychiatric disorders like schizophrenia and MDD have been primarily treated using pharmacotherapy such as with antipsychotic or antidepressant medications, both of which have been shown to reduce psychiatric symptoms (Leucht et al., 2012; Undurraga and Baldessarini, 2012). However, several large-scale meta-analyses have recently suggested that the effectiveness of antipsychotic and antidepressant medications is only marginally different from that of a placebo (Leucht et al., 2009; Rief et al., 2009), and neither have been able to successfully remediate cognitive dysfunction (Keefe et al., 2013, 2014; Solé et al., 2015). These approaches are generally not designed to target neuroplasticity, but rather to remediate other aspects of a psychiatric disorder such as dysfunctional neurotransmitter systems. Neither antipsychotics nor antidepressants have been consistently shown to induce any lasting neuroplastic changes in the brain (Rief et al., 2015). With the growing importance that is being placed on deficits in neuroplasticity to the underlying etiology of psychiatric disorders, it may be necessary to stimulate underlying neuroplastic changes in order to induce lasting structural alterations and effectively alleviate cognitive dysfunctions.

For example, there have been equivocal findings regarding the impact of antipsychotic medications like haloperidol (typical) and olanzapine (atypical) on macro-scale hippocampal structure, with studies finding chronic use to cause volumetric increases (Schmitt et al., 2004), decreases (Barr et al., 2013), and no impact at all (Navari and Dazzan, 2009; Smieskova et al., 2009; Vernon et al., 2011). Micro-scale changes associated with the chronic use of antipsychotics have also been mixed, for example it remains unclear whether any antipsychotic has a consistent impact on hippocampal neurogenesis (Schoenfeld and Cameron, 2015). Even in cases where antipsychotics like olanzapine were found to increase the number of adult-born cells in the DG, these cells were likely to be endothelial cells and oligodendrocytes rather than granule cells (Kodama et al., 2004), which may not promote DG function and mechanisms like pattern separation in the same way. The impact of both types of antipsychotics on synaptic plasticity is also ambiguous, with some studies showing drugs like olanzapine to promote dendritic growth (Park et al., 2013), while others have found that various typical and atypical antipsychotics are associated with a general reduction in dendritic complexity and impaired LTP (Frost et al., 2010; Price et al., 2014). Finally, BDNF is considered to be an important factor underlying the pathophysiology of schizophrenia. However, typical antipsychotics have been largely associated with reductions in BDNF expression and the impact of atypical antipsychotics remains unclear, with many studies finding it to have no impact on BDNF expression in the hippocampus and across other regions (Favalli et al., 2012).

Furthermore, no antipsychotic medication (Goldberg et al., 2007; Tybura et al., 2013) or other pharmacological intervention (Keefe et al., 2013) has been consistently demonstrated to alleviate the cognitive deficits associated with schizophrenia and in cases where the medication is anticholinergic, cognitive deficits have been shown to worsen (Tandon, 2011). The most promising approach in restoring cognitive deficits in schizophrenia may in fact, be through cognitive remediation which has produced some significant improvements in a number of cognitive domains and functional outcomes (McGurk et al., 2007; Reddy et al., 2014), however, effect sizes remain only small to moderate (Wykes et al., 2011). Currently, cognitive deficits are one of the most debilitating aspects of schizophrenia that remain the least effectively treated (Gibbons and Dean, 2016).

In the case of treating MDD, widely prescribed antidepressant medications such as selective serotonin reuptake inhibitors (SSRIs) have been more convincingly demonstrated to induce neuroplastic changes. SSRI medications have been shown to stimulate the rate of hippocampal neurogenesis (Malberg et al., 2000; Boldrini et al., 2009), but again this may not be representative of increased granule cells. It has been suggested that this effect is likely due to a ‘dematuration’ of mature granule cells in the DG rather than increased cell proliferation and it is unclear what impact this would have on hippocampal functioning (Kobayashi et al., 2010). For example, mature granule cells are intrinsic to pattern completion (Nakashiba et al., 2012) so this dematuration of granule cells caused by SSRIs could lead to an underactivation of pattern separation and inhibit proper episodic memory formation. However, various antidepressant treatments have reliably been shown to up-regulate hippocampal BDNF (Duman and Monteggia, 2006; Musazzi et al., 2009), which appears to be promoting synaptic plasticity within the region (Bath et al., 2012). Despite this, antidepressant medications are encumbered with slow response rates, a modest therapeutic efficacy and little or no impact on the cognitive deficits associated with MDD (Duman and Aghajanian, 2012; McIntyre et al., 2013; Rosenblat et al., 2015; Solé et al., 2015; Bortolato et al., 2016). Despite the increasing importance placed on alleviating cognitive dysfunction in MDD, research has continued to focus on treating affective symptoms (Baune and Renger, 2014). Subsequently, there are currently no prescribed treatments available that effectively alleviate cognitive deficits in MDD (Keefe et al., 2014; Solé et al., 2015; Bortolato et al., 2016).

Through directly targeting neuroplasticity in crucial areas like the hippocampus, it may be possible to induce lasting structural changes that promote the region’s functioning and contribute toward the alleviation of cognitive dysfunction in psychiatric disorders. With the global pharmaceutical industry being estimated to reach a value of US$1.3 trillion by 2018 (CMR International, 2015), bridging the gap between neuroplasticity and functional outcomes in treating psychiatric disorders will likely be dominated by a pharmacological approach. Indeed, a great amount of research is being conducted into developing an effective pharmacological intervention to treat cognitive deficits in psychiatric disorders (Wallace et al., 2011). However, none of the most promising pharmacological agents have been able to achieve more than a moderate effect size in treating most cognitive deficits, at least within MDD (Keefe et al., 2014; Solé et al., 2015) and schizophrenia (Keefe et al., 2013). Therefore, it may be worth considering the inclusion of non-pharmacological approaches such as AE, as an adjunction to pharmacotherapy that may improve treatment of cognitive dysfunction.

### The Prospect of an AE Intervention

While its therapeutic potential has only been explored very recently, AE has been demonstrated to counteract pathologically induced hippocampal harm and improve the region’s functioning in a range of animal disease models including fetal alcohol spectrum disorders, traumatic brain injury, stroke, and Parkinson’s, Alzheimer’s and Huntington’s diseases (Patten et al., 2015). In humans, AE has been shown to stimulate hippocampal neuroplasticity and successfully counteract deteriorating hippocampal function caused by aging or Alzheimer’s disease (Intlekofer and Cotman, 2013). However, whether these findings can be extended to individuals with psychiatric disorders is still unclear. Most of the existing human literature investigating the use of AE in the treatment of MDD or schizophrenia has only measured its impact on individual psychopathologies, such as depressive symptoms or positive and negative symptoms (Knöchel et al., 2012). In both cases, AE has been demonstrated to be effective in reducing both positive and negative symptoms of schizophrenia (Vancampfort et al., 2012; Firth et al., 2015; Rimes et al., 2015) and affective symptoms in MDD (Cooney et al., 2012), in some cases just as effectively as antidepressants (Blumenthal et al., 1999, 2007; Brosse et al., 2002), or in terms of neurogenesis, exceeding it (Marlatt et al., 2010). The efficacy of AE in reducing a range of psychiatric symptoms suggests that AE interventions could have a number of benefits to the treatment of disorders like MDD or schizophrenia. Importantly, AE appears to be able to stimulate neuroplasticity and promote hippocampal functioning in brains with both healthy and pathologically deteriorated hippocampi. It is plausible that AE interventions could be used to counteract hippocampal harm caused by disorders that profoundly impact upon hippocampal functioning, and through this approach, aid in the alleviation of certain aspects of cognitive dysfunction.

In recent years, AE has started to attract attention as a therapeutic target for schizophrenia treatment, but only a handful of studies have systematically investigated the capacity for an AE intervention to remediate cognitive deficits associated with the disorder (Knöchel et al., 2012; Sommer and Kahn, 2015). Both cross-sectional (Kimhy et al., 2014) and interventional studies (Pajonk et al., 2010; Oertel-Knöchel et al., 2014; Kimhy et al., 2015; McEwen et al., 2015) have demonstrated that AE can promote cognitive functioning in patients with schizophrenia across a range of cognitive domains including speed of processing, short-term and working memory and visual learning. Although one review was unable to find an association between AE and a reduction of cognitive symptoms in patients with schizophrenia (Dauwan et al., 2016) these early results are largely promising.

On a cellular level, some studies have used animal models of schizophrenia to demonstrate that AE can promote neurogenesis (Wolf et al., 2011), hippocampal BDNF concentration and the expression of NDMA receptors in the hippocampus (Kim T.W. et al., 2014; Park et al., 2014). One study using an animal model of schizophrenia also demonstrated AE to improve performance on a spatial working memory task (Kim T.W. et al., 2014). These initial results suggest that AE could be beneficial for improving hippocampal structure and functioning in schizophrenia but such findings must be interpreted with caution given the aforementioned difficulty of modeling complex disorders like schizophrenia in animals (Nestler and Hyman, 2010). Some recent studies in human patients with schizophrenia have shown AE interventions to be associated with increased BDNF serum concentrations (Kuo et al., 2013; Kim H. et al., 2014; Kimhy et al., 2015) and one study found that the increased BDNF concentration accounted for a significant proportion of the improvements they had observed in cognitive performance observed in following AE (Kimhy et al., 2015). Another study in patients with schizophrenia also demonstrated AE to counteract the deterioration of white-matter tract integrity that is associated with the disorder (Svatkova et al., 2015).

These findings are encouraging and some human imaging studies have suggested the positive impact of AE may be region-specific to the hippocampus. For example, in patients with schizophrenia a 12-week AE intervention has led to increased hippocampal volume, hippocampal NAA concentration and improved performances on short-term memory and working memory tasks (Pajonk et al., 2010; Lin et al., 2015; McEwen et al., 2015). However, other studies have found AE interventions to of had no impact on hippocampal volume or function in patients with schizophrenia (Scheewe et al., 2013; Rosenbaum et al., 2015). It is possible that these discrepancies are caused by systemic differences between the studies. Although data was not available for all studies (Rosenbaum et al., 2015) attendance rates for AE sessions were generally higher in those studies that did find improvements in hippocampal volume and function (Pajonk et al., 2010; Lin et al., 2015) than those which did not (Scheewe et al., 2013). Additionally, both studies that found no volume change used an automated algorithm to segment the hippocampus (Scheewe et al., 2013; Rosenbaum et al., 2015), which is known to be less accurate than the manual method (Morey et al., 2009) employed in the former study (Pajonk et al., 2010) – although this was not always the case (Lin et al., 2015).

In patients with MDD, several interventional studies have demonstrated that AE can promote cognitive performance in executive functioning, attention, inhibitory control, speed of processing, working and spatial memory and visual learning (Kubesch et al., 2003; Vasques et al., 2011; Oertel-Knöchel et al., 2014; Greer et al., 2015). Although one study did not find AE to have any positive impact on cognition (Hoffman et al., 2008), the results so far are promising and AE may represent a viable option in remediating cognitive dysfunction in MDD (Solé et al., 2015).

In animal models of stress/MDD, certain micro-scale changes have been recorded which demonstrate that AE can promote hippocampal neurogenesis, vascularization, BDNF expression (both brain-wide and within the hippocampus), IGF-1 expression, VEGF expression, and hippocampal synaptic plasticity (Adlard and Cotman, 2004; Zheng et al., 2006; Bjørnebekk et al., 2010; Nakajima et al., 2010; Sartori et al., 2011; Kiuchi et al., 2012; Yau et al., 2012; Lu et al., 2014). Animal models of stress/MDD have also shown that AE can reduce depressive-like behavioral and deficits in spatial memory (Zheng et al., 2006; Nakajima et al., 2010; Yau et al., 2012). In studies of human patients with MDD, AE has been associated with increased BDNF serum concentration (Gustafsson et al., 2009; Laske et al., 2010). Just one human study has sought to assess macro-scale structural changes and found no association between an AE intervention and hippocampal volume or neurotrophin circulation (Krogh et al., 2014). However, participant attendance rate to AE sessions was very low in this study, at less than half (one session per week) the attendance rate recorded by studies in other psychiatric populations who did demonstrate growth in hippocampal volume (2.6 sessions per week; Pajonk et al., 2010). Furthermore, participants in this study also showed no reduction in depressive symptoms. This indicates that there may not have been a sufficient level of engagement in AE for changes in hippocampal structure and neurobiology to be recorded.

In addition to promoting neuroplastic changes that could lead to cognitive enhancement, AE is also thought to interact with several neurotransmitter systems, including the monoamine system (Chaouloff, 1989). The action of the monoamine neurotransmitter serotonin (5-hydroxytryptamine, 5-HT) in the hippocampus is known to facilitate the process of hippocampal learning and memory (Riedel et al., 1999; Buhot et al., 2000). 5-HT transmission in the hippocampus is disrupted in schizophrenia and MDD (Naughton et al., 2000; Middlemiss et al., 2002) and this 5-HT dysfunction is likely to contribute deficits in learning and memory in these disorders (Meeter et al., 2006; Gray and Roth, 2007). The exact nature of this dysfunction is unclear given the diverging impact that agonists and antagonists have on each 5-HT receptor subtype within the hippocampus and the complex interactions between 5-HT and other neurotransmitter systems (Meneses, 1999). Targeting specific hippocampal 5-HT receptors, particularly the 5-HT_1a_ receptor subtype, is growing in popularity as a pharmacological approach designed to promote learning and memory in psychiatric disorders (Wallace et al., 2011) like schizophrenia (Meltzer and Sumiyoshi, 2008) or MDD (Meeter et al., 2006). Some animal studies have indicated that AE may also be associated with elevated levels of 5-HT in the hippocampus (Gomez-Merino et al., 2001) as well as an increase in tryptophan hydroxylase, a rate-limiting precursor of 5-HT produced in the raphe nucleus, which projects directly to the hippocampus (Chaouloff, 1989). It is unclear whether this interaction with hippocampal 5-HT would be beneficial or not, but some recent animal work has indicated that AE can specifically ameliorate dysfunction in the 5-HT_1a_ receptor subtype (Maniam and Morris, 2010; Kim M.H. et al., 2014). The impact of AE on the monoamine system has largely been discussed in terms of its antidepressant properties (Christie et al., 2008; van Praag, 2009), but it is possible that this interaction may also contribute toward cognitive enhancement. However, more work is needed to elucidate the exact role of 5-HT and 5-HT receptor subtypes in hippocampal learning and memory and whether this coincides with the actions of AE.

The inclusion of AE interventions into current treatment approaches could be useful to counteract hippocampal harm and alleviate cognitive dysfunctions caused by psychiatric disorders. The important role that AE could play in the treatment of cognitive dysfunction is starting to gain traction in relation to schizophrenia (Malchow et al., 2013; Sommer and Kahn, 2015; Vakhrusheva et al., 2016) and MDD (Malchow et al., 2013; Oertel-Knöchel et al., 2014). Despite this, there is a distinct lack of systematic investigations (both animal and human) into the relationship between AE and neuroplasticity, hippocampal functioning, and its impact on cognitive dysfunctions in psychiatric disorders such as MDD (Malchow et al., 2013) or schizophrenia (Sommer and Kahn, 2015). Based on the available evidence, the merits of AE are best capitulated as an adjunctive intervention to pharmacotherapy that could improve treatment efficacy through targeting cognitive dysfunctions that remain largely untreated. It is possible that pharmacotherapy may eventually develop a comprehensive treatment for cognitive dysfunction, but non-pharmacological approaches like AE have a number of distinct benefits to the patient (discussed below), which make these interventions worthy of further investigation by future research.

## Why Are Non-Pharmacological Interventions Useful?

The efficacy of both antidepressant and antipsychotic medications could be improved should more attention be afforded to relevant lifestyle factors such as AE engagement (Rief et al., 2015). There appears to be a degree of overlap in the underlying mechanisms that are stimulated by AE and certain pharmacological medications, such as with the antidepressant fluoxetine (Huang et al., 2012). It is possible that such a degree of overlap in their underlying mechanisms could mean that combining a traditional pharmacological intervention with an AE intervention would have a synergising impact on inducing neuroplasticity. For example, animal models have demonstrated that the combination of AE and antidepressant treatment had a stronger impact on up-regulating BDNF than either intervention had individually (Russo-Neustadt et al., 2001; Baj et al., 2012) – this is particularly important as BDNF regulation is thought to be crucial to the antidepressant mechanism (Duman and Aghajanian, 2012). Additionally, the inclusion of AE to a traditional antidepressant intervention has been shown to have greater impact on reducing depressive symptoms in patients with MDD, than antidepressant treatment alone (Knubben et al., 2007; Schuch et al., 2011; Legrand and Neff, 2016). Interestingly, MDD patients with higher basal levels of BDNF due to prior treatment with SSRI’s experienced a more rapid reduction in symptoms following an AE intervention than those with lower basal BDNF levels (Toups et al., 2011). This suggests that antidepressant medications may be useful for ‘priming’ patients with MDD to respond better to a subsequent AE intervention (Toups et al., 2011). Additionally, in animals, AE was significantly more potent at increasing the survival of adult-born granule cells in comparison to SSRIs like fluoxetine and duloxetine (Marlatt et al., 2010). Therefore, the inclusion of AE to a traditional antidepressant intervention could also have an additive impact on promoting neurogenesis.

The use of AE as an adjunctive treatment to traditional antidepressant medication may have a synergising impact on neuroplasticity potentially resulting in a more effective approach toward remediating psychiatric symptoms. The possibility of an enhanced efficacy rate would be particularly useful in treating patients with MDD who do not respond to antidepressant treatment alone (Mura et al., 2014), which could be as many as 10-30% of patients with MDD (Joffe et al., 1996). An interesting direction for future research would be to investigate whether including AE as an adjunctive treatment to a traditional pharmacological approach would have a synergistic impact on neuroplasticity, resulting in a greater treatment efficacy than attainable by either intervention alone.

In addition to the potential benefits that a combined approach may have on symptom alleviation, the inclusion of AE interventions may have further benefits to the well being of patients. For example, the development of a psychiatric disorder significantly increases the risk of psychiatric comorbidity (Fusar-Poli et al., 2014; Avenevoli et al., 2015). AE is conversely associated with lowering the risk of various other conditions developing that range from age- and dementia-related cognitive decline to mood and anxiety disorders (Martinsen, 2008; Ahlskog et al., 2011; Mammen and Faulkner, 2013). For example, a recent systematic review concluded that AE was effective at reducing the risk of patients with schizophrenia developing a comorbid disorder (Firth et al., 2015). The inclusion of AE interventions could be useful in reducing the risk of patients developing other comorbidities, but AE may be of an even greater utility in helping to reduce the risk of pharmacologically induced side effects.

### Side Effects in Pharmacology

Pharmacological treatments are generally associated with a higher risk of inducing adverse side effects than non-pharmacological interventions in patients of MDD or schizophrenia (Gartlehner et al., 2015, 2016). Both antipsychotic (De Hert et al., 2012) and antidepressant (Anderson et al., 2012) medications are associated with numerous adverse side effects that range from more common and less severe symptoms like headaches or nausea, to less common but more severe symptoms like cardiovascular or metabolic dysfunction. Independent of influences from medication, psychiatric populations are already at an elevated risk of cardiovascular, metabolic or respiratory dysfunction than the general population (Galletly et al., 2012; Vancampfort et al., 2013) and this risk may be heightened further by certain pharmacological treatments. Contrastingly, AE is known to elicit comparatively few adverse side effects and is associated with improvements in the social, physical and affective well being of individuals with psychiatric disorders (Fiuza-Luces et al., 2013). Moreover, AE is known to be preventative of cardiovascular, metabolic and respiratory dysfunction, which suggests that the inclusion of an AE intervention may be useful in stemming the risk of psychiatric patients developing these severe conditions (Caemmerer et al., 2012; Vancampfort et al., 2014). Some evidence of this can be drawn from recent animal studies that have demonstrated that AE ameliorates the metabolic, lipid peroxidation and extrapyramidal side effects of antipsychotic medication (Czéh et al., 2007; Teixeira et al., 2011; Baptista et al., 2013; Boyda et al., 2014).

Concerns are also mounting over the growing length of time in which a patient is subjected to pharmacotherapy, as the long-term impact of these medications is unclear. There is currently a distinct lack of longitudinal studies that systematically evaluate the impact of long-term treatment with widely used antidepressants like SSRI’s on brain and behavior (Popovic et al., 2015). Some studies have suggested that long-term antidepressant treatment (generally lasting more than 6 months) can have a detrimental impact on executive function, memory, attention, and motivation in patients with MDD (Fava et al., 2006; Popovic et al., 2015; Bortolato et al., 2016). Worryingly, one recent animal study demonstrated that the long-term administration of fluoxetine at clinically relevant doses was associated with impaired dendritic spine morphology leading to deficits in hippocampal synaptic plasticity (Rubio et al., 2013). This is particularly concerning given the increasing importance that is being placed on the role of hippocampal synaptic plasticity in the effective treatment of MDD (Duman and Aghajanian, 2012). Evidence is also accumulating to suggest that long-term antipsychotic treatment in humans may be linked to a reduction in both global gray- and white-matter volumes (Navari and Dazzan, 2009; Ho et al., 2011; Vernon et al., 2011; Fusar-Poli et al., 2013) as well as region-specific reductions in hippocampal volume (Panenka et al., 2007).

There is an insufficient amount of data to make any definitive statements about the long-term impact these pharmacological interventions could be having on the brain. However, evidence may continue to emerge that shows the long-term application of widely used antidepressants and antipsychotics as having a detrimental impact on neuroplasticity and cognitive performance. The inclusion of AE interventions could be useful in counterbalancing some of these harms, at least with regard to the hippocampus, through the direct impact that AE has on promoting neuroplasticity and cognition. For example, recent animal studies have demonstrated that AE was able to reverse most of damage caused to hippocampal volume and hippocampal synaptic connectivity caused by antipsychotic medication (Barr et al., 2013; Ramos-Miguel et al., 2015). In addition, the aforementioned potential for a combined pharmacotherapy/AE approach to have an additive impact on symptom alleviating may mean that patients reach remission at a faster rate. Thereby, the inclusion of AE interventions may also reduce the total time a patient is exposed to pharmacotherapy, lowering their risk of any adversities associated with long-term use.

Developing further pharmacological interventions that effectively alleviate cognitive symptoms in medicated psychiatric populations will undoubtedly provide a more comprehensive treatment approach, but it could also elevate the risk of adverse side effects for the patient. AE has the potential to directly combat these adverse side effects as well as contributing toward the remediation of cognitive deficits, as well as various other psychiatric symptoms that may be related to hippocampal dysfunction. AE interventions are unlikely to represent a viable standalone treatment for cognitive dysfunction, but may make an important, low-risk adjunctive treatment to pharmacotherapy that promotes the effectiveness and reduces the harms associated with pharmacological interventions. However, this approach must be considered in light of some limitations associated with AE intervention.

### Limitations of AE Interventions

Psychiatric patients often view pharmacological interventions more negatively than by the doctors who prescribe them (Nosè et al., 2012) and it is possible that patients may perceive a non-pharmacological adjunctive approach more favorably. Psychiatric patients have previously reported having a favorable perspective of AE treatment (Stanton and Reaburn, 2014), however, this is not necessarily reflected in actual AE engagement (Vancampfort et al., 2016). Motivating psychiatric patients to engage in regular AE is likely to be the greatest obstacle in both researching and implementing AE interventions in treatment, as epitomized by the high dropout rates in AE interventional studies in psychiatric samples (Stubbs et al., 2016; Vancampfort et al., 2016). Amongst those who are physically able to exercise, the lack of motivation is a significant factor preventing individuals with schizophrenia (Soundy et al., 2014) or MDD (Krämer et al., 2014) from adhering to AE interventions. It is possible that certain pharmacological treatments may contribute to this issue as psychiatric patients have previously reported medication side effects as a primary factor that inhibits their capacity to engage in regular exercise (Glover et al., 2013). While many studies investigating AE interventions in psychiatric patients do now incorporate motivational strategies (e.g., motivational interviews or goal setting) to encourage AE engagement, motivational factors are rarely included as a primary outcome measure (Farholm and Sørensen, 2016). Increasingly, efforts are being concentrated on promoting adherence to AE interventions in psychiatric patients (Knapen et al., 2015). For example, the integration of action video games with AE interventions has been shown as a promising method of enhancing the adherence of patients with schizophrenia to improving their aerobic fitness (Kimhy et al., 2016b). However, more research must be dedicated to directly studying the relationship between motivation and AE in psychiatric populations in order to measure and improve the effectiveness of motivational strategies.

Another issue in the conceptualization of AE treatments is the lack of consensus as to what type, intensity or length of exercise sessions has the strongest impact on the brain (Prakash et al., 2015). For example, some studies argue that a high-intensity exercise is optimal for reducing symptoms in MDD (Singh et al., 2005), while others have suggested that that a mild (Dunn et al., 2005) or a moderate intensity exercise intervention would be optimal (Stanton and Reaburn, 2014). It is possible that exercise intensities may vary depending on the purpose of the intervention, for example it has been suggested that improving cognitive performance may require high-intensity, interval training but preserving cognitive function in an aging brain may require a lower intensity, more continuous protocol (Duzel et al., 2016). Exercise type may also be an important factor. Although most of the current literature has focussed on AE, other forms of exercise such as yoga (Lin et al., 2015) or weight training (Suo et al., 2016) may also be beneficial in promoting brain health and cognition. Given the growing interest in exercise as a therapeutic intervention, it is surprising that only a handful of studies have attempted to systematically establish the most effective way in which it should be applied in psychiatric populations (Perraton et al., 2010; Stanton and Happell, 2014; Stanton and Reaburn, 2014; Kimhy et al., 2016a). It is important that future research concentrates on establishing the merits of different forms of exercise and fully outlining the dose-response relationship between the intensity and length of AE intervention and therapeutic outcome in each psychiatric population.

## Conclusion

Research demonstrating the potential for AE to promote hippocampal structure and function is growing at an impressive rate as more and more work is translated from animal to human models. Importantly, the beneficial impact that AE has on the brain may have a useful clinical application in treating disorders in which hippocampal damage is a significant factor that underlies its symptomatology. There is currently a particular need to develop effective strategies that alleviate cognitive dysfunction and targeting deficits in the neuroplasticity of crucial areas to cognition like the hippocampus, is a promising approach to remediating cognitive dysfunction. AE interventions represent an effective method of promoting hippocampal neuroplasticity and function that encompasses few risks and several additional benefits to the patient, such as combating pharmacologically induced side effects. This paper has highlighted two promising examples of how AE interventions could improve the treatment of schizophrenia and MDD, but AE interventions could well have a broader application in mental health such as in treating substance abuse (Zschucke et al., 2012).

However, several issues must be addressed for AE to be successfully implemented as an adjunct to pharmacotherapy. Firstly, more RCTs are needed to systematically establish a causal relationship between AE with neural and cognitive outcomes. Future RCTs could benefit from a more hippocampus-focussed approach with particular regard to the choice of cognitive tasks used in the study. The use of techniques like multimodal imaging and peripheral biomarker assays should also be incorporated to build a comprehensive account of the impact that AE has on the brain. A greater focus should be placed on investigating the impact of AE in psychiatric populations both in terms of neuroplastic changes and therapeutic efficacy. An interesting avenue of research is to assess to what extent AE could interact with current pharmacological treatments to reduce side effects and have a synergistic impact on neuroplasticity and symptom reduction. Finally, future research should strive to establish standardized methodologies for investigating AE and the most effective method in which an AE intervention would be implemented to maximize therapeutic outcome. Improving our understanding of the role that lifestyle factors such as exercise play in maintaining and promoting brain functioning could have major implications for the way in which we treat, or even prevent, psychiatric and neurological disorders.

## Author Contributions

AK was responsible for the conception and writing of the review and received substantial, direct and intellectual contributions from JH, PL, and MY. All authors approved the paper for publication.

## Conflict of Interest Statement

The authors declare that the research was conducted in the absence of any commercial or financial relationships that could be construed as a potential conflict of interest.
